# Twenty-year trend in mortality among hospitalized patients with pneumococcal community-acquired pneumonia

**DOI:** 10.1371/journal.pone.0200504

**Published:** 2018-07-18

**Authors:** Catia Cillóniz, Adamantia Liapikou, Ignacio Martin-Loeches, Carolina García-Vidal, Albert Gabarrús, Adrian Ceccato, Daniel Magdaleno, Josep Mensa, Francesc Marco, Antoni Torres

**Affiliations:** 1 Department of Pneumology, Institut Clinic del Tórax, Hospital Clinic of Barcelona—Institut d'Investigacions Biomèdiques August Pi i Sunyer (IDIBAPS), University of Barcelona (UB)—SGR 911- Ciber de Enfermedades Respiratorias (Ciberes), Barcelona, Spain; 2 Respiratory Department, Sotiria Chest Diseases Hospital, Mesogion, Athens, Greece; 3 Multidisciplinary Intensive Care Research Organization (MICRO), St James's University Hospital, Department of Clinical Medicine, Trinity College, Dublin, Ireland; 4 Department of Infectious Disease, Hospital Clinic of Barcelona, Barcelona, Spain; 5 Superior Medical School of the National Polytechnic Institute Mexico City, Mexico City, Mexico; 6 Department of Microbiology, Biomedical Diagnostic Center (CDB), ISGlobal, Barcelona Center for International Health Research (CRESIB), Hospital Clinic of Barcelona, University of Barcelona, Barcelona, Spain; Azienda Ospedaliero Universitaria Careggi, ITALY

## Abstract

**Background:**

There is only limited information on mortality over extended periods in hospitalized patients with pneumococcal community-acquired pneumonia (CAP). We aimed to evaluate the 30-day mortality and whether is changed over a 20-year period among immunocompetent adults hospitalized with pneumococcal CAP.

**Methods:**

We conducted a retrospective observational study of data that were prospectively collected at the Hospital Clinic of Barcelona of all adult patients hospitalized with diagnosis of pneumococcal CAP over a 20-year period. To aid analysis, results were divided into four periods of 5 years each (1997–2001, 2002–2006, 2007–2011, 2012–2016). The primary outcome was 30-day mortality, but secondary outcomes included intensive care unit (ICU) admission, lengths of hospital and ICU-stays, ICU-mortality, and need of mechanical ventilation.

**Results:**

From a cohort of 6,403 patients with CAP, we analyzed the data for 1,120 (17%) adults with a diagnosis of pneumococcal CAP. Over time, we observed decreases in the rates of alcohol consumption, smoking, influenza vaccination, and older patients (age ≥65 years), but increases in admissions to ICU and the need for non-invasive mechanical ventilation. The overall 30-day mortality rate was 8% (95% confidence interval, 6%–9%; 84 of 1,120 patients) and did not change significantly between periods (p = 0.33). Although, we observed a decrease in ICU-mortality comparing the first period (26%) to the second one (10%), statistical differences disappeared with adjustment (p0.38).

**Conclusion:**

Over time, 30-day mortality of hospitalized pneumococcal CAP did not change significantly. Nor did it change in the propensity-adjusted multivariable analysis. Since mortality in pneumococcal pneumonia has remained unaltered for many years despite the availability of antimicrobial agents with proven in vitro activity, other non-antibiotic strategies should be investigated.

## Introduction

Community-acquired pneumonia (CAP) continues to have high global morbidity and mortality rates, and is associated with considerable health costs[1,2]. Some recent published studies have reported that the incidence of CAP has risen in the last decade[3,4], with an increase in the rate of hospitalization. However, the trend in CAP mortality over recent decades is more controversial, and it was recently stated that mortality in patients hospitalized for CAP has declined over time[5–7]. Many clinical guidelinesfor CAP recommended by professional societies have been published since the early 1990s [8]. The aim of creating the guidelines was to a large extent to bring together all the data on CAP and to provide a tool for the management of CAP patients, since guidelines offer a means of standardizing care. The first set of guidelines for CAP was published by ATS in 1993[8]; other guidelines were subsequently published, updating the information with the results of new published studies on CAP, including IDSA 1998[9], ATS 2001[10], and the ATS/IDSA Joint Guidelines in 2007[11]. Two previous studies have demonstrated reductions in mortality after guideline implementation: one in hospitalized CAP[12]] and the other in severe CAP[13]. In fact, mortality has declined for a variety of conditions over recent decades, including for sepsis, myocardial infarction, and stroke[14,15], suggesting that better clinical management and improved healthcare are responsible[5–7].

*Streptococcus pneumoniae* (pneumococcus) remains the most common pathogen in CAP[16,17], being associated with significant disease burden and a more severe disease course that requires more medical resources compared with non-pneumococcal CAP[18]. In Catalonia, the study setting, vaccination with 23-valent polysaccharide pneumococcal vaccine (PPV23) has been recommended for high-risk and older adults since the early 2000s[19]. The 7-valent pneumococcal conjugate vaccine (PCV7)was licensed in Spain for children In 2001, and it was replaced by the 13-valent pneumococcal conjugate vaccine (PCV13) in 2010[20]. In Catalonia in 2007 the estimated coverage of PCV7 was 47%, and the estimated coverage of PCV13 in 2013 was 55%[21,22]. However, except in the case of children with selected risk factors these vaccines were not financed by the Catalan Public Health System until July 2016, and were only available in the private sector. In Spain, the vaccines were not introduced into the recommended calendar until January 2017. Finally, in late 2012, the PCV13 was introduced for adults. A recent population-based cohort study involving more than two million people aged over 50 in Catalonia which investigated the clinical effectiveness of PCV13 vaccination in preventing hospitalization from pneumonia observed no clinical benefits from PCV13 vaccination. However, since in that study PCV13 coverage was low, vaccination was not randomized, and follow-up time was limited, the results must be interpreted with caution[23].

In a large homogeneous cohort of critically ill patients with pneumococcal CAP, Mongardon et al.[24] observed that septic shock occurred in 84% and that mechanical ventilation was required for 77%. The authors highlighted that, despite adequate antimicrobial therapy, pneumococcal pneumonia still has high early mortality (29%).

We hypothesized that mortality among patients hospitalized with pneumococcal CAP had decreased over time because of improvements in early detection, antibiotic therapy, clinical management, support bundles and improvement of health care systems. Thus, we aimed to analyze 30-day mortality rate and the trend in mortality for pneumococcal CAP in a large hospitalized adult population over a 20-year period. We also analyzed the main factors related to overall mortality from pneumococcal CAP.

## Patients and methods

### Study design

This was a retrospective observational study of data that were prospectively collected over 20 years in the Hospital Clinic, Barcelona. All adults with a clinical diagnosis of *S*. *pneumoniae* pneumonia between January 1996 and December 2016 were included. To aid analysis, we divided the study population into four cohorts by time period: 1997–2001, 2002–2006, 2007–2011, and 2012–2016. For the purpose of publication the Ethics Committee of the Hospital Clinic of Barcelona approved the study (Register: 2009/5451), and the need for written informed consent was waived because of the non-interventional design.

### Patients

We included all adults with a clinical diagnosis of pneumococcal pneumonia, including those coming from nursing homes. We excluded patients with active tuberculosis or immunosuppression, including those taking >20 mg/day of prednisone (or daily equivalent) for at least 2 weeks, those receiving cytotoxic therapy, those known to have human immunodeficiency virus infection.

### Data collection, evaluation, and microbiological diagnosis

Demographic variables, comorbidities, and physiological parameters were collected in the emergency department within 24 hours of admission. We also calculated the pneumonia severity index (PSI), the CURB-65 (Consciousness, Urea, Respiratory rate, Blood pressure, 65 years old), and the sequential organ failure assessment (SOFA) scores at admission[25–27]. During hospitalization, we recorded whether the patients had complications such as multilobar infiltration, pleural effusions, acute respiratory distress syndrome(ARDS)[28]], septic shock[29], or acute renal failure[30]. Further details are reported elsewhere[17].

The etiology of pneumococcal pneumonia was determined by positive results in any of the following: valid sputum culture; valid blood culture; pleural fluid and transthoracic needle aspiration cultures; urinary antigen for *S*. *pneumoniae*; bacterial growth exceeding 10^3^, 10^4^, or 10^5^ colony forming units (CFU)/mL in protective brush sample, bronchoalveolar lavage cultures or tracheobronchial aspirate, respectively. Definition and additional details are shown in Supplementary Material.

### Primary and secondary outcomes

The primary outcome was 30-day mortality, and the secondary outcomes were the lengths of hospital and ICU stays, admission to ICU, mortality in ICU, and need for mechanical ventilation. Early mortality was defined as the mortality in the first 72 hours of admission and late mortality was defined as the mortality after 72 hours to 30 days of admission.

### Statistical analysis

We report the number and percentage of patients for categorical variables, the mean and standard deviation (SD) for normally distributed data, and the median and interquartile range (IQR) for non-normally distributed data. To reduce the variability and random noise in year-by-year data we divided the study into four time periods of five years each, defining 1997–2001 as the reference period. We estimated the overall 30-day mortality and the mortality in each of the four periods, reporting them with 95% confidence intervals (CIs)[31]. Trends in associated factors were analyzed using the Mantel–Hansel test for categorical variables and linear regression for continuous variables. Time to 30-day mortality was analyzed by Kaplan–Meier survival curves and compared using the Gehan–Breslow–Wilcoxon test. Cox proportional hazard regression analyses[32] were performed to determine the influence of risk factors on the 30-day mortality. First, each risk factor was tested individually (see Methods in Supplementary Material the full list of variables). Second, a propensity score (PS)[33] was developed for empiric antibiotic use because antibiotic therapy were not randomly administered and could have resulted in confounding and selection bias[34]. Finally, the scores, admission period, and empiric antibiotic therapy were incorporated in the multivariable Cox regression model to predict 30-day mortality, together with all risk factors showing an association in the univariable analyses by stepwise backward elimination (p_in_ < 0.05, p_out_ > 0.10). We calculated the hazard ratios (HRs) and their 95% CIs. We calculated the area under the receiver operating characteristic curve (AUC) for the multivariable model to predict 30-day mortality. Internal validity of the prediction model was assessed by ordinary nonparametric bootstrapping with 1000 bootstrap samples and bias-corrected, accelerated 95% CIs[35]. Also, to determine the influence of the risk factors on ICU mortality, the analyses were repeated on the subset of patients admitted to ICU.

We used multiple imputation method for missing data for handling missing data in multivariable analyses[36]. The level of significance was set at 0·05 (two-tailed). All analyses were performed with IBM SPSS Statistics 23·0 (Armonk, New York).

## Results

### Patients’ characteristics

**Fig 1:**
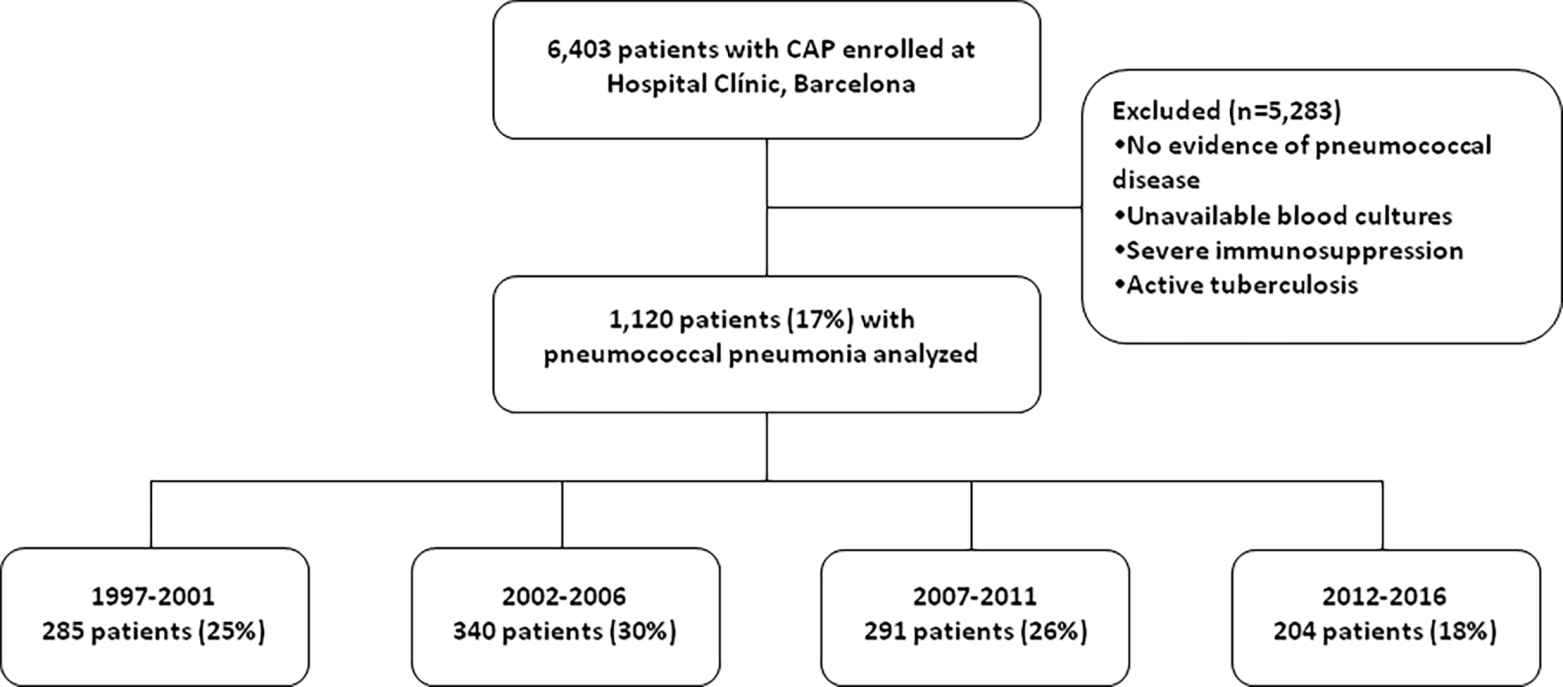
Flow chart of study population.

Of the 6,403 patients with CAP admitted during the study period, 1,120 (18%) were diagnosed with pneumococcal pneumonia and included in the study “Fig 1”. Our cohort comprised 694 males (62%) and 426 females (38%), with a mean (SD) age of 66.9 (17.7) years; notably, 701 (63%) were aged ≥65 years.

### Changes in patients’ characteristics over time

The main clinical characteristics of the study population are summarized by study period in “Table 1”. There were no differences in sex between periods, but there were differences in the distribution of cases by age, smoking status, and alcohol consumption, which were significantly higher in the first period compared with later periods.

**Table 1:** Patients’ characteristics by study period.

Variable	1997–2001 (n = 285)	2002–2006 (n = 340)	2007–2011 (n = 291)	2012–2016 (n = 204)	P-value for trend
Age, mean (SD), years	67.4 (16.4)	66.9 (18.0)	65.9 (18.2)	67.6 (18.4)	0.82
Age ≥65 years, n (%)	194 (68)	215 (63)	168 (58)	124 (61)	**0.029**
Male sex, n (%)	205 (72)	186 (55)	173 (60)	130 (64)	0.095
Current smoker, n (%)	99 (35)	96 (28)	80 (28)	47 (23)	**0.006**
Current alcohol consumer, n (%)	72 (26)	60 (18)	47 (17)	30 (15)	**0.002**
Previous antibiotic, n (%)	31 (11)	61 (18)	41 (16)	32 (16)	0.18
Influenza vaccine, n (%)	20 (32)	127 (42)	118 (47)	49 (25)	**0.030**
Pneumococcal vaccine, n (%)	11 (18)	45 (15)	40 (16)	36 (19)	0.51
Previous inhaled corticosteroids, n (%)	57 (20)	67 (20)	77 (27)	33 (17)	0.87
Previous systemic corticosteroids, n (%)	12 (12)	27 (8)	9 (3)	10 (5)	**0.004**
Previous episode of pneumonia (last year), n (%)	28 (11)	71 (21)	26 (10)	14 (7)	**0.012**
Comorbidities, n (%)^a^	221 (78)	240 (71)	187 (64)	131 (64)	**<0.001**
Chronic respiratory disease	172 (61)	160 (47)	127 (45)	65 (33)	**<0.001**
COPD	138 (49)	98 (29)	74 (26)	39 (20)	**<0.001**
Asthma	9 (3)	24 (7)	16 (6)	7 (4)	0.88
Bronchiectasis	9 (3)	7 (2)	11 (4)	1 (1)	0.28
Other^b^	16 (6)	31 (9)	26 (9)	18 (9)	0.15
Chronic cardiovascular disease	47 (17)	52 (15)	41 (14)	11 (6)	**0.001**
Diabetes mellitus	55 (19)	78 (23)	41 (14)	40 (20)	0.46
Neurological disease	32 (11)	42 (13)	46 (17)	35 (18)	**0.016**
Chronic renal disease	23 (8)	17 (5)	21 (7)	17 (9)	0.64
Chronic liver disease	30 (11)	23 (7)	15 (5)	16 (8)	0.15
Nursing-home, n (%)	9 (3)	16 (5)	24 (8)	10 (5)	0.093
Creatinine, mg/dL, median (IQR)	1.1 (0.9; 1.4)	1.2 (1; 1.6)	1 (0.8; 1.5)	1.2 (0.9; 1.7)	0.47
C-reactive protein, mg/dL, median (IQR)	169 (85; 296)	221 (111; 317)	242 (147; 308)	246 (148; 290)	0.59
PaO_2_/FiO_2_, median (IQR)	276 (229; 314)	281 (243; 319)	267 (218; 301)	266 (203; 308)	**0.002**
SOFA score, median (IQR)	2 (2; 4)	3 (2; 4)	2 (1; 3)	2 (1; 3)	**<0.001**
SOFA score ≥5, n (%)^c^	53 (19)	54 (16)	35 (12)	12 (8)	**0.002**
PSI score, median (IQR)	102 (80; 127)	100 (77; 126)	100 (75; 120)	105 (80; 125)	0.43
PSI risk class IV–V, n (%)^d^	173 (62)	188 (60)	107 (55)	59 (64)	0.57
CURB-65 risk class 3–5, n (%)^e^	74 (26)	88 (26)	51 (20)	31 (21)	0.090
Pneumococcal bacteremia, n (%)	104 (39)	113 (41)	93 (37)	70 (41)	0.90
Invasive pneumococcal pneumonia, n (%)	111 (41)	121 (42)	101 (39)	73 (42)	0.98
Pleural effusion, n (%)	49 (17)	59 (17)	68 (24)	30 (16)	0.64
Multilobar, n (%)	75 (26)	97 (29)	76 (26)	66 (32)	0.28
ARDS, n (%)	12 (4)	13 (4)	19 (7)	16 (8)	**0.026**
Acute renal failure, n (%)	80 (28)	115 (34)	83 (29)	72 (36)	0.21
Septic shock, n (%)	27 (9)	34 (10)	50 (17)	31 (15)	**0.005**
Empiric antibiotic therapy, n (%)					
Monotherapy	50 (18)	81 (24)	60 (21)	33 (16)	0.63
Fluoroquinolones	8 (3)	68 (20)	50 (17)	19 (9)	**0.025**
β-lactams	34 (12)	11 (3)	10 (3)	12 (6)	**0.003**
Other therapy	8 (3)	2 (1)	0 (0)	2 (1)	**0.020**
Combination therapies	232 (82)	259 (76)	230 (79)	169 (84)	0.63
β-lactams plus macrolides	185 (66)	148 (44)	87 (30)	97 (48)	**<0.001**
β-lactams plus fluoroquinolones	4 (1)	84 (25)	119 (41)	57 (28)	**<0.001**
Other combination therapies	43 (15)	27 (8)	24 (8)	15 (7)	**0.005**
Appropriate empiric treatment, n (%)	274 (97)	328 (97)	280 (97)	198 (99)	0.63

Abbreviations: ARDS indicates acute respiratory distress syndrome; COPD = chronic obstructive pulmonary disease; CURB-65 = Consciousness, Urea, Respiratory rate, Blood pressure, 65 years old; IQR, interquartile range; PSI, pneumonia severity index; PaO_2_/FiO_2_ = arterial oxygen tension to inspired oxygen fraction ratio; SD, standard deviation; SOFA, sequential organ failure assessment. Percentages calculated on non-missing data.

^a^ May have >1 comorbid condition.

^b^ Other respiratory diseases include sequelae of pulmonary tuberculosis, pulmonary hypertension, and interstitial lung disease.

^c^ Optimal cut-off value to predict 30-day mortality using ROC curves.

^d^ Stratified according to 30-day risk mortality for community-acquired pneumonia: risk classes I–III (≤90 points) have low mortality and risk classes IV–V (>90 points) have the highest mortality.

^e^ Stratified according to 30-day risk mortality for community-acquired pneumonia: risk classes 0–2 have low mortality and risk classes 3–5 have the highest mortality.

Regarding severity, there was a progressive increase in presentations with ARDS (4% in the first period to 8% in the last period; p = 0.026) and patients with septic shock (9% in the first period to 15% in the last period; p = 0.005).

The prevalence of pneumococcal CAP did not change significantly over time, only varying between 39% and 42% (p = 0.98) “Table 2”. From 2002, 167 of 420 invasive isolates (40%) were available for serotyping, and the percentages of cases covered by the pneumococcal conjugate vaccine (PCV13) decreased from 94% in the second period to 56% in the last period (p = 0.001). Regarding empiric antibiotic therapy, we observed no differences in prescriptions of monotherapy and combination therapy between periods. The adequacy of antibiotic therapy was similar among periods (97%).

**Table 2:** Samples for microbiological diagnosis of pneumococcal pneumonia by study period.

Microbiological test	1997–2001 (n = 285)	2002–2006 (n = 340)	2007–2011 (n = 291)	2012–2016 (n = 204)	P-value for trend
Urinary antigen test for pneumococcus	111/120 (93)	222/255 (87)	199/238 (84)	131/168 (78)	**0.001**
Blood culture	104/268 (39)	113/279 (41)	93/252 (37)	70/170 (41)	0.90
Pleural fluid culture	12/37 (32)	8/32 (25)	13/48 (27)	7/23 (30)	0.81
Sputum culture	131/189 (69)	61/197 (31)	59/155 (38)	35/96 (37)	**0.001**
Bronchoalveolar aspirate	26/42 (62)	8/26 (31)	16/42 (38)	6/19 (32)	**0.005**

Data are shown as number of patients with pneumonia due to *Streptococcus pneumoniae* (pneumococcus) / number of patients for each microbiological examination performed (%).

### Clinical outcomes

The most frequent causes of death were acute respiratory failure secondary to pneumonia (54%; n = 45), multiorgan failure associated with septic shock (36%; n = 30), and acute cardiac events related to pneumonia (8%; n = 7).

The overall 30-day mortality rate among all 1,120 patients was 8% (95% CI, 6%–9%; n = 84). By subgroup, the 30-day mortality rates were as follows. In the 701 patients aged ≥65 years, it was 10% (95% CI, 8%–12%; n = 69) compared with 4% (95% CI, 2%–5%; n = 15) in the 419 patients aged <65 years (p < 0.001). In the 406 cases with invasive pneumococcal CAP it was 10% (95% CI, 7%–13%; n = 40) compared with 6% (95% CI, 4%–8%; n = 36) in the 579 patients with non-invasive pneumococcal CAP (p = 0.035). Finally, the 30-day mortality among 815 patients hospitalized to conventional wards was 4% (95% CI, 2%–5%; n = 30), but this increased to 18% (95% CI, 13%–22%; n = 53) among the 302 patients admitted to ICU (p < 0.001).

The 30-day mortality rate also changed with the number of comorbidities. In 779 patients with at least one comorbidity, the rate was 9% (95% CI, 7%–11%; n = 71) compared with 4% (95% CI, 2%–6%; n = 13) among 338 healthy patients (p = 0.002). As expected, compared with the 30-day mortality of 7% (95% CI, 5%–9%; n = 75) among the 1,080 patients receiving adequate antibiotic therapy, mortality increased to 25% (95% CI, 10%–40%; n = 8) among the 32 patients receiving inadequate antibiotic therapy (p < 0.001).

The frequency of mortality in the first 72 hours of admission (1.4% in the first period to 1.5% in the last period; p 0.89) and the frequency of mortality after 72 hours to 30 days (7% in the first period to 8% in the last period; p 0.33) did not change over time. Outcomes by study period are shown in “Table 3”.

**Table 3:** Outcomes by study period.

Variable	1997–2001 (n = 285)	2002–2006 (n = 340)	2007–2011 (n = 291)	2012–2016 (n = 204)	P-value for trend
30-day mortality, n (%)	24 (8)	16 (5)	24 (8)	20 (10)	0.33
Length of hospital stay, median (IQR), days	8 (5; 11)	8 (5; 12.5)	9 (6; 12)	8 (6; 13)	0.070
ICU admission, n (%)	57 (20)	78 (23)	100 (34)	67 (33)	**<0.001**
ICU mortality, n (%)^a^	15 (26)	8 (10)	11 (11)	7 (10)	**0.023**
Length of stay in ICU, median (IQR), days^a^	12 (8; 24)	14 (9; 27)	11 (8; 17.5)	12 (8; 19)	0.20
Mechanical ventilation, n (%)^b^					0.78
Not ventilated	249 (87)	292 (89)	217 (84)	139 (86)	0.31
Non-invasive	0	14 (4)	17 (7)	11 (7)	**<0.001**
Invasive	36 (13)	23 (7)	26 (10)	11 (7)	0.11

Abbreviations: ICU, intensive care unit; IQR, interquartile range. Percentages calculated on non-missing data.

^a^ 57 patients in the 1997–2001 period, 78 patients in the 2002–2006 period, 100 in the 2007–2011 period, and 67 patients in the 2012–2016 period were used to calculate the percentages and the medians (IQR).

^b^ Patients who initially received non-invasive ventilation but subsequently needed intubation were included in the invasive mechanical ventilation group.

**Fig 2:**
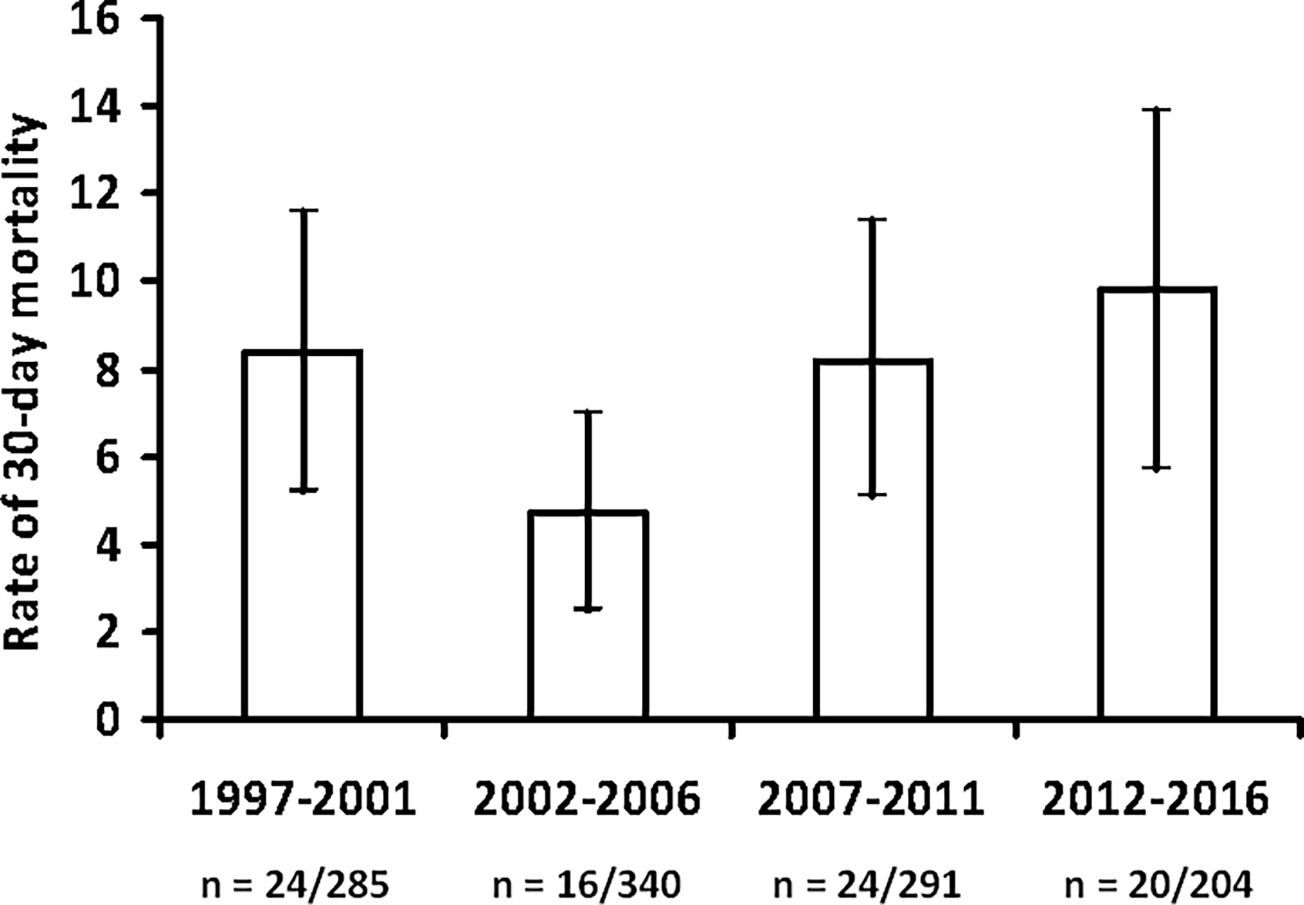
Rate of 30-day mortality per period.

**Fig 3:**
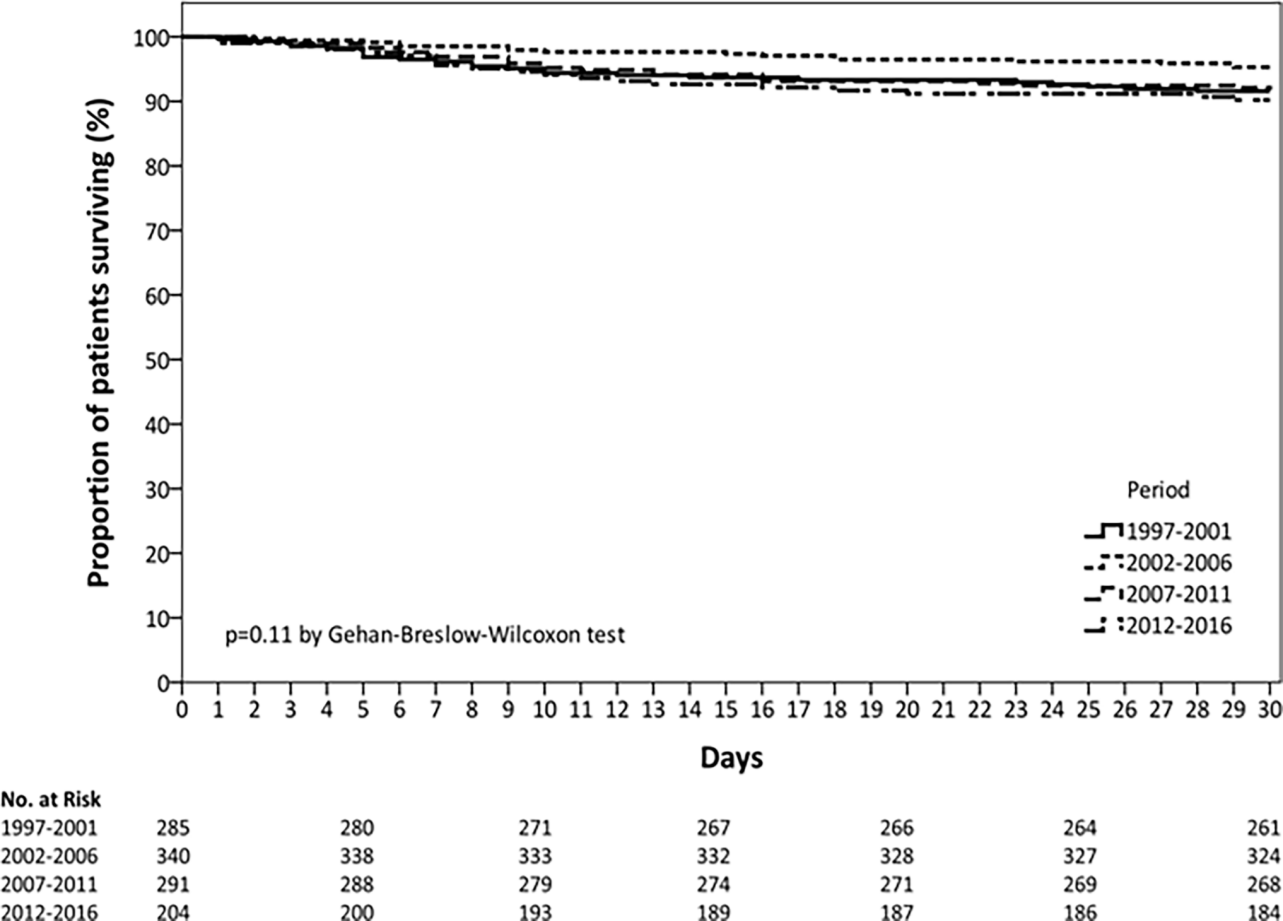
Kaplan-Meier analysis on the effect of period on time to death.

The overall 30-day mortality rate did not change significantly between periods (p = 0.33), ranging from 8% in the first period to 10% in the last period “Fig 2”. Kaplan–Meier survival curves depicting 30-day mortality rates as a function of period are shown in “Fig 3”. There was a progressive increase in patients requiring ICU admission (20% in the first period to 33% in the last period; p < 0.001) and non-invasive mechanical ventilation (NIV) (0% in the first period to 7% in the last period; p < 0.001). The use of invasive mechanical ventilation did not change between the four periods. ICU mortality was 26% in the first period, it dropped to 10% in the second period, and remained stable thereafter (p = 0.023). However, after adjusted by propensity-adjusted multivariable analysis, study period was not associated with ICU mortality.

In the subgroup of patients receiving NIV, ICU mortality changed non-significantly from 0% in the first period to 9% in the last period (p = 0.23). By Kaplan–Meier analysis, time to death in the ICU and time to death of patients receiving NIV in the ICU did not differ significantly between periods (p = 0.17 and p = 0.76, respectively) (S1 Fig).

### Factors associated with 30-Day mortality and ICU mortality

The univariable Cox regression analysis revealed several variables to be significantly associated with 30-day mortality “Table 4”. Among these, age ≥65 years, diabetes mellitus, SOFA score ≥5, and requirement for mechanical ventilation (non-invasive and invasive) were independently associated with 30-day mortality in the propensity-adjusted multivariable analysis. Period of admission was not significantly associated with 30-day mortality after adjustment for potential confounders.

**Table 4:** Significant univariable and multivariable Cox regression analyses for the prediction of 30-day mortality.

Variable	Univariable^a^			Multivariable^b^		
	HR	95% CI	P-value	HR	95% CI	P-value
Period of admission^c^			0.12			0.12
1997–2001	1.00	–	–	1.00	–	–
2002–2006	0.54	0.29 to 1.02	0.060	1.67	0.15 to 18.95	0.68
2007–2011	0.97	0.55 to 1.71	0.93	4.03	0.20 to 81.23	0.36
2012–2016	1.17	0.65 to 2.12	0.60	3.09	0.46 to 20.97	0.25
Age ≥65 years	2.83	1.62 to 4.95	<0.001	2.93	1.63 to 5.27	<0.001
Previous systemic corticosteroids	1.51	0.94 to 2.42	0.090	–	–	–
Chronic renal disease	2.58	1.43 to 4.67	0.002	–	–	–
Chronic liver disease	2.10	1.14 to 3.87	0.017	–	–	–
Neurologic disease	1.61	1.00 to 2.60	0.052	–	–	–
Diabetes mellitus	2.07	1.26 to 3.39	0.004	1.68	0.99 to 2.85	0.054
SOFA score ≥5^d^	7.22	4.70 to 11.08	<0.001	3.91	2.17 to 7.03	<0.001
Invasive pneumococcal pneumonia	1.63	1.04 to 2.55	0.034	–	–	–
Empiric antibiotic therapy^e^			0.001			0.11
Beta-lactams monotherapy	1.14	0.49 to 2.63	0.76	1.08	0.44 to 2.63	0.87
Fluoroquinolones monotherapy	0.51	0.22 to 1.19	0.12	1.52	0.59 to 3.94	0.38
Beta-lactams plus fluoroquinolones	0.96	0.51 to 1.81	0.90	1.32	0.62 to 2.81	0.48
Beta-lactams plus macrolides	0.35	0.18 to 0.68	0.002	0.59	0.29 to 1.21	0.15
Other	1.00	–	–	1.00	–	–
Adequate empiric antibiotic therapy	0.22	0.11 to 0.45	<0.001	–	–	–
ICU admission	5.17	3.31 to 8.02	<0.001	–	–	–
Mechanical ventilation^f^			<0.001			<0.001
Not ventilated	1.00	–	–	1.00	–	–
Non-invasive	5.86	2.69 to 12.78	<0.001	2.95	1.22 to 7.16	0.017
Invasive	8.85	5.58 to 14.02	<0.001	4.07	2.40 to 6.88	<0.001

Abbreviations: CI indicates confidence interval; HR, hazard ratio; ICU, intensive care unit; SOFA, sequential organ failure assessment. Data are shown as estimated HRs (95% CIs) of the explanatory variables in the 30-day mortality group. The HR is the ratio of hazards (probability of death) in two groups, given that the patient has survived up to a specific time. The P-value is based on the null hypothesis that all HRs relating to an explanatory variable equal unity (no effect).

^a^ The variables analyzed in the univariable analysis were: age, gender, tobacco use, alcohol consumption, influenza and pneumococcal vaccination, previous systemic and inhaled corticosteroids, prior antibiotic treatment, chronic pulmonary disease, chronic cardiovascular disease, chronic renal disease, chronic liver disease, diabetes mellitus, neurological disease, SOFA score, invasive disease pneumonia, adequate empiric antibiotic therapy, ICU admission, and mechanical ventilation.

^b^ Adjusted for the propensity score.

^c^ The p-value corresponds to differences between the four groups (1997–2001, 2002–2006, 2007–2011, or 2012–2016).

^d^ Optimal cut-off value to predict 30-day mortality using ROC curves.

^e^ The p-value corresponds to differences between the five groups (beta-lactams monotherapy, fluoroquinolones monotherapy, beta-lactams plus fluoroquinolones, beta-lactams plus macrolides, or other).

^f^ The p-value corresponds to differences between the three groups (not ventilated, non-invasive, or invasive).

The AUC was 0.70 (95% CI, 0.64–0.76) for the model predictive of 30-day mortality (S2 Fig). The sensitivity was 63% (95% CI, 52%–74%), specificity was 68% (95% CI, 65%–71%), positive predictive value was 14% (95% CI, 10%–17%), negative predictive value was 96% (95% CI, 94%–97%), positive likelihood ratio was 1.97 (95% CI, 1.64–2.38), and negative likelihood ratio was 0.54 (95% CI, 0.41–0.72). Internal validation of the Cox regression model by bootstrapping demonstrated robust results for all variables included in the model, with small 95% CIs around the original coefficients (S1 Table).

The univariable Cox regression analysis revealed several variables significantly associated with ICU mortality among the subset of patients admitted to the ICU “Table 5”. Notably, age ≥65 years and requirement for invasive mechanical ventilation were independently associated with ICU mortality in the propensity-adjusted multivariable analysis. Again, however, period of admission was not significantly associated with ICU mortality after adjustment for potential confounders. The AUC was 0.70 (95% CI, 0.62–0.78) for the model predictive of ICU mortality (S3 Fig). The sensitivity was 85% (95% CI, 73%–97%), specificity was 49% (95% CI, 43%–55%), positive predictive value was 21% (95% CI, 14%–27%), negative predictive value was 96% (95% CI, 92%–99%), positive likelihood ratio was 1.67 (95% CI, 1.40–1.99), and negative likelihood ratio was 0.30 (95% CI, 0.14–0.63). Internal validation of the Cox regression model by bootstrapping demonstrated robust results for all the variables included in the model, with small 95% CIs around the original coefficients (S2 Table).

**Table 5:** Significant univariable and multivariable cox regression analyses for the prediction of icu mortality.

Variable	Univariable^a^			Multivariable^b^		
	HR	95% CI	P-value	HR	95% CI	P-value
Period of admission^c^			0.10			0.38
1997–2001	1.00	–	–	1.00	–	–
2002–2006	0.36	0.15 to 0.85	0.020	2.72	0.04 to 211.36	0.65
2007–2011	0.54	0.25 to 1.19	0.13	7.56	0.03 to 1,688.48	0.46
2012–2016	0.50	0.20 to 1.23	0.13	1.96	0.06 to 67.07	0.71
Age ≥65 years	2.34	1.17 to 4.67	0.016	2.27	1.08 to 4.79	0.031
SOFA score ≥5^d^	2.68	1.40 to 5.13	0.003	–	–	–
Empiric antibiotic therapy^e^			0.21			0.29
Beta-lactams monotherapy	1.66	0.45 to 6.14	0.45	0.75	0.18 to 3.04	0.68
Fluoroquinolones monotherapy	1.13	0.31 to 4.19	0.85	2.84	0.63 to 12.76	0.17
Beta-lactams plus fluoroquinolones	0.58	0.25 to 1.35	0.21	0.98	0.33 to 2.90	0.97
Beta-lactams plus macrolides	0.50	0.21 to 1.19	0.12	0.58	0.23 to 1.42	0.23
Other	1.00	–	–	1.00	–	–
Adequate empiric antibiotic therapy	0.37	0.17 to 0.79	0.011	–	–	–
Mechanical ventilation^f^			0.001			0.017
Not ventilated	1.00	–	–	1.00	–	–
Non-invasive	2.07	0.58 to 7.33	0.26	2.21	0.60 to 8.23	0.24
Invasive	4.97	2.04 to 12.08	<0.001	3.76	1.51 to 9.37	0.005

Abbreviations: CI indicates confidence interval; HR, hazard ratio; ICU, intensive care unit; SOFA, sequential organ failure assessment. Data are shown as estimated HRs (95% CIs) of the explanatory variables in the ICU mortality group. The HR is the ratio of hazards (probability of death) in two groups, given that the patient has survived up to a specific time. The P-value is based on the null hypothesis that all HRs relating to an explanatory variable equal unity (no effect).

^a^ The variables analyzed in the univariable analysis were: age, gender, tobacco use, alcohol consumption, influenza and pneumococcal vaccination, previous systemic and inhaled corticosteroids, prior antibiotic treatment, chronic pulmonary disease, chronic cardiovascular disease, chronic renal disease, chronic liver disease, diabetes mellitus, neurological disease, SOFA score, invasive disease pneumonia, adequate empiric antibiotic therapy, and mechanical ventilation.

^b^ Adjusted for the propensity score.

^c^ The p-value corresponds to differences between the four groups (1997–2001, 2002–2006, 2007–2011, or 2012–2016).

^d^ Optimal cut-off value to predict 30-day mortality using ROC curves.

^e^ The p-value corresponds to differences between the five groups (beta-lactams monotherapy, fluoroquinolones monotherapy, beta-lactams plus fluoroquinolones, beta-lactams plus macrolides, or other).

^f^ The p-value corresponds to differences between the three groups (not ventilated, non-invasive, or invasive).

## Discussion

There are three main findings to this study. First, it was striking that the 30-day mortality of patients hospitalized for pneumococcal CAP did not change significantly over time (this included early and late mortality). Second, although we observed a progressive increase in the number of patients admitted to ICU and receiving NIV between periods, the mortality of patients receiving NIV in ICU did not change over that time. Third, mortality in ICU fell from 26% in the first study period to 10% in the second period, and remained stable thereafter; however, when we adjusted by propensity-adjusted multivariable analysis, study period was not associated with higher mortality.

Recently, there have been few studies about the changes in CAP-related mortality over time[5,6,37], with only limited information about the 30-day mortality in patients hospitalized with pneumococcal CAP over study periods of a decade or longer[24].

This study is the first, to our knowledge, to describe trends in 30-day mortality over a 20 year period in patients hospitalized with confirmed pneumococcal CAP. It was remarkable that we found no evidence of 30-day mortality for pneumococcal CAP decreasing in hospitalized adult patients over that period. Nevertheless, our results are consistent with those of epidemiological research in Canada (excluding Quebec) over a 6 year period[24]. In that study, the authors reported that case-fatality rates from pneumococcal pneumonia remained stable (11.6% to 12.3%) between 2004 and 2010, although they did report a decrease in the incidence of CAP due to *S*. *pneumoniae*, which they attributed to vaccination programs. However, they relied entirely on retrospective data extracted from an administrative database, which would be subject to potential biases, especially in the case of *S*. *pneumoniae* diagnosis. By contrast, our data acquisition was prospective and relied on a confirmed microbiological diagnosis of *S*. *pneumoniae*. In addition, our 30-day mortality was 3%–4% lower than that reported by the Canadian group.

Although we did not observe a fall in 30-day mortality over time, an interesting finding was the decrease in the presence of comorbidities, and also the decrease in the proportion of current smokers over time. One possible explanation is the change of pneumococcal serotypes over the course of the study period. Since the beginning of 2000, serotype evolution in pneumococcus has been in constant progress, influenced by the introduction of pneumococcal conjugate vaccines (PCVs)[21,38]. Despite the effectiveness of the PCV in reducing the burden of invasive pneumococcal disease, the incidence of complicated pneumonia[39] is rising because of the emergence of non-vaccine serotypes. This increase does not appear to be related to the concurrent increase in penicillin-resistant *S*. *pneumoniae* but does seem related to the introduction of virulent clones expressing non-vaccine serotypes, especially serotype 1[40]. On the other hand, we cannot rule out the possible effect of ambient air pollution on on the incidence of pneumococcal infections[41–43] as reported by a Spanish study of 619 cases of pneumococcal infections which found that fossil fuel-derived pollutants (SO_2_, NO), and dry and cold air increased the incidence of pneumococcal infections[43].

We observed an increase in patients admitted to ICU, as well increases in the proportions of patient with ARDS, with septic shock, and requiring NIV. These data are consistent with results of a UK study in which it was reported that a growing number of CAP cases have required ICU admission in the last 20 years, regardless of age groups[44]. The reason for the increase in ICU admission is probably related to a better knowledge among clinicians of the definitions of severe CAP in international guidelines[11,45].

ICU mortality was 26% in the first period, dropped to 10% in the second period, and then remained stable. However, we could not confirm this downward trend and subsequent stabilization when adjusting the results by propensity-adjusted multivariable analysis. We think that the reduction in ICU mortality between the first and the second periods of our study was related to the 2001 and 2007 CAP guidelines[10,11], recommendations from the Surviving Sepsis campaign[29], and the implementation of early initiation of the first antibiotic dose[46]. There are two previous studies, one in hospitalized CAP[12] and another one in severe CAP[13], that have demonstrated reductions in mortality after guideline implementation.

It is noteworthy that our data are in partial disagreement with the results of Gattarello et al.[37], who conducted a matched case-control study (80 cases and 80 controls) comparing the periods 2000–2002 and 2008–2013. They reported a 15% decrease in mortality among patients with pneumococcal pneumonia admitted to the ICU, and that early antibiotic administration and combination antibiotic therapy were independently associated with better outcomes. However, there were important differences with our study: (a) we included the data for 1,120 patients and the study by Gattarello et al.[37] included only 160 patients; (b) more than 60% of the patients (n = 701) were aged ≥65 years in our cohort, whereas only 33% (n = 54) were aged ≥65 years in their cohort; and c) antibiotic therapy did not affect mortality in our study because most patients received adequate empirical antibiotic therapy.

The major strengths of our study are the large cohort, long study period, consecutive enrollment, and inclusion of only cases with microbiologically confirmed pneumococcal CAP. However, a potential limitation of the study is that data were collected from a single academic teaching hospital in Spain, which has extensive experience in the management of CAP; consequently, it may not be possible to extrapolate our results to patients admitted to other types of hospitals in other countries. Other potential limitations are the changes in the diagnosis, treatment, and prevention of CAP during the 20 years of study for which we did not account. However, our antibiotic treatment protocol has not changed substantially for CAP during these years, and we have followed the 1993, 1998, 2001, and 2007 guidelines for managing CAP[8–11].

In conclusion, we observed increases in the rates of patients admitted to ICU and in the rates of ARDS, septic shock, and requirement for NIV. We did not find a decrease in mortality from pneumococcal CAP. Given that mortality from pneumococcal pneumonia has not changed for many years despite treatment with appropriate antimicrobial therapy, there is a growing need for non-antibiotic strategies to be investigated.

## Supporting information

S1 Fig Kaplan–Meier analysis of the effect of period on time to death in intensive care. (TIF)

S2 Fig ROC Analysis of significant variables derived from the logistic regression model to predict 30-day mortality. (TIF)

S3 Fig ROC analysis of significant variables derived from the logistic regression model to predict ICU mortality. (TIF)

S1 Table Internal validation of the prediction model for 30-Day mortality by the nonparametric bootstrap technique. (DOCX)

S2 Table Internal validation of the prediction model for ICU mortality by the nonparametric bootstrap technique. (DOCX)
