# Supplementary figures and images for: Twenty-year trend in mortality among hospitalized patients with pneumococcal community-acquired pneumonia
Source: PLoS One. 2018 Jul 18;13(7):e0200504. doi: 10.1371/journal.pone.0200504 (PMC6051626; doi:10.1371/journal.pone.0200504)

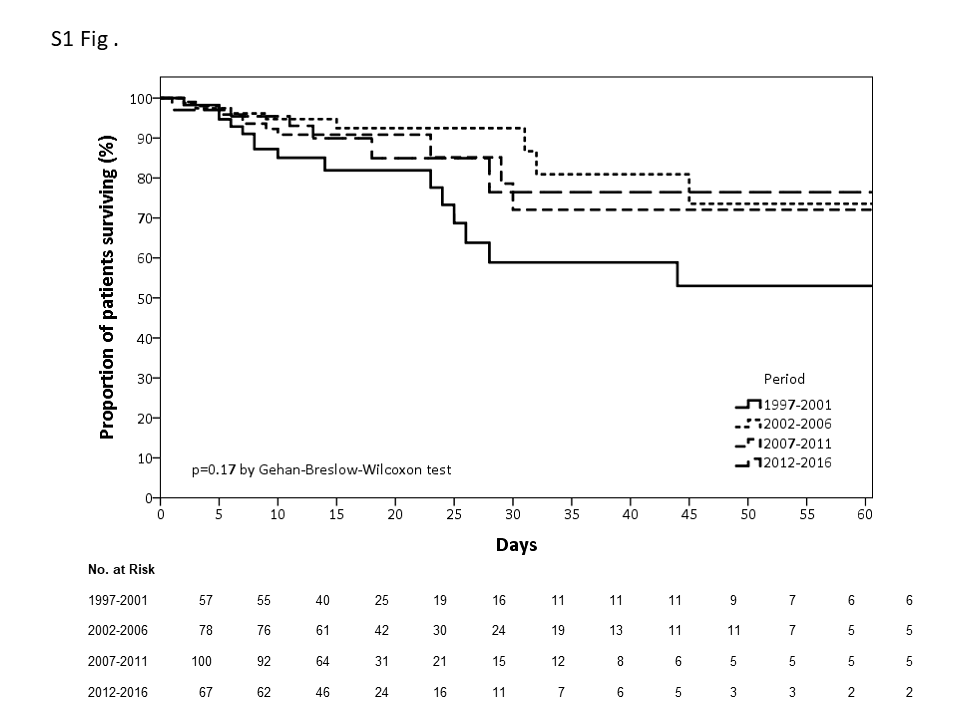

Supplement: S1 Fig — (TIF) [file pone.0200504.s001.tif]

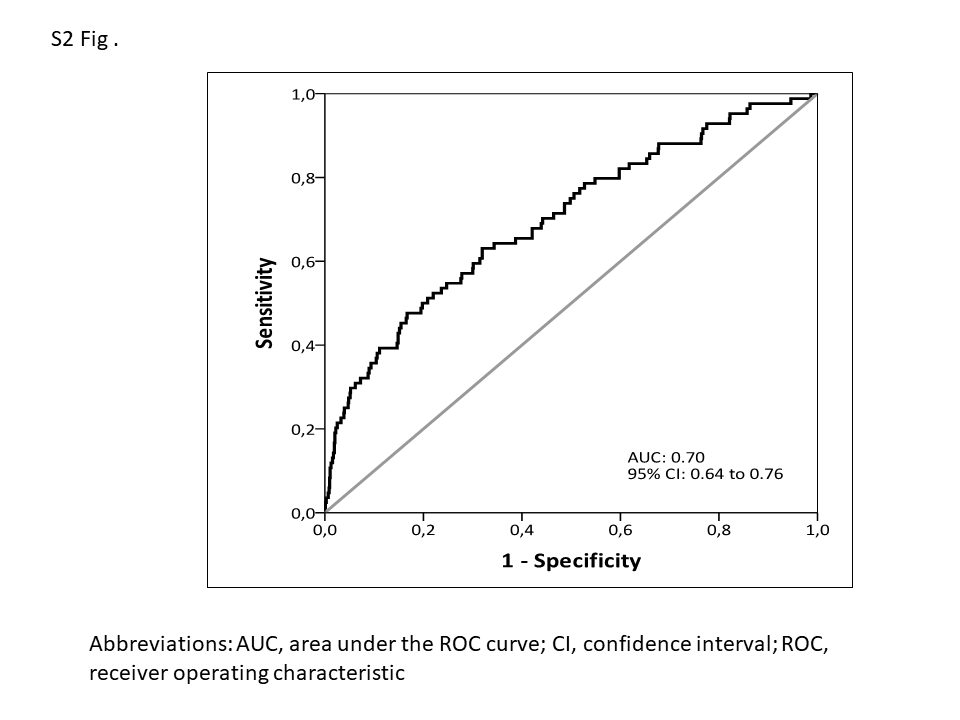

Supplement: S2 Fig — (TIF) [file pone.0200504.s002.tif]

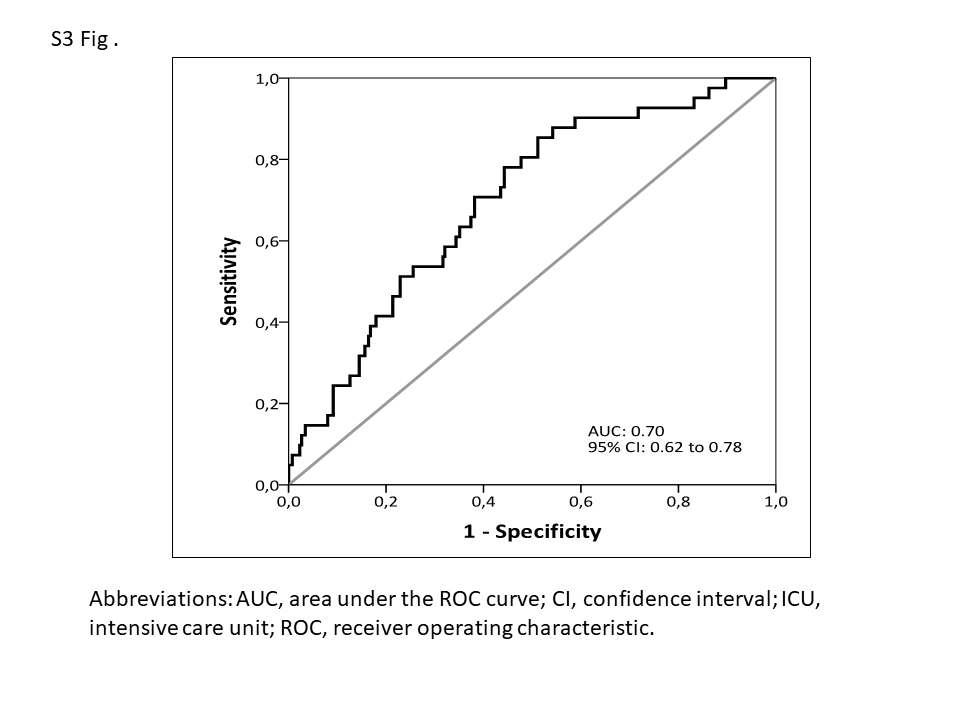

Supplement: S3 Fig — (TIF) [file pone.0200504.s003.tif]
