# Supplementary material for: Twenty-year trend in mortality among hospitalized patients with pneumococcal community-acquired pneumonia
Source: PLoS One. 2018 Jul 18;13(7):e0200504. doi: 10.1371/journal.pone.0200504 (PMC6051626; doi:10.1371/journal.pone.0200504)
Supplement: S1 Table — (DOCX) [file pone.0200504.s004.docx]

S1 Table.

| **Variable** | **Original** | **Bias** | **SE** | **95% BCa CI** |
| --- | --- | --- | --- | --- |
| Period of admission 2002-2006 | 0.512 | 0.069 | 1.477 | -2.491 to 3.428 |
| Period of admission 2007-2011 | 1.393 | 0.096 | 1.779 | -2.098 to 5.068 |
| Period of admission 2012-2016 | 1.127 | 0.088 | 1.069 | -1.063 to 3.481 |
| Age ≥65 years | 1.074 | 0.070 | 0.304 | 0.507 to 1.899 |
| Diabetes mellitus | 0.519 | -0.010 | 0.282 | -0.094 to 1.053 |
| SOFA score ≥5 | 1.363 | 0.049 | 0.311 | 0.684 to 2.134 |
| Beta-lactams monotherapy | 0.076 | 0.000 | 0.522 | -0.940 to 1.041 |
| Fluoroquinolone monotherapy | 0.421 | -0.007 | 0.504 | -0.572 to 1.349 |
| Βeta-lactams plus fluoroquinolones | 0.275 | 0.015 | 0.439 | -0.568 to 1.190 |
| Βeta-lactams plus macrolides | -0.521 | -0.016 | 0.402 | -1.277 to 0.228 |
| Non-invasive mechanical ventilation | 1.081 | -0.036 | 0.497 | 0.013 to 1.963 |
| Invasive mechanical ventilation | 1.403 | 0.005 | 0.302 | 0.721 to 1.986 |

Abbreviations: BCa, adjusted bootstrap; CI, confidence interval; SE, standard error; SOFA, sequential organ failure assessment.
