# Supplementary material for: Twenty-year trend in mortality among hospitalized patients with pneumococcal community-acquired pneumonia
Source: PLoS One. 2018 Jul 18;13(7):e0200504. doi: 10.1371/journal.pone.0200504 (PMC6051626; doi:10.1371/journal.pone.0200504)
Supplement: S2 Table — (DOCX) [file pone.0200504.s005.docx]

S2 Table.

| **Variable** | **Original** | **Bias** | **SE** | **95% BCa CI** |
| --- | --- | --- | --- | --- |
| Period of admission 2002-2006 | 1.001 | 0.403 | 3.199 | -6.109 to 8.727 |
| Period of admission 2007-2011 | 2.023 | 0.572 | 3.968 | -6.631 to 12.138 |
| Period of admission 2012-2016 | 0.672 | 0.267 | 2.473 | -4.696 to 6.565 |
| Age ≥65 years | 0.821 | 0.078 | 0.454 | -0.036 to 2.130 |
| Beta-lactams monotherapy | -0.293 | -0.597 | 3.112 | -13.389 to 1.803 |
| Fluoroquinolone monotherapy | 1.044 | -0.326 | 2.661 | -11.552 to 3.451 |
| Βeta-lactams plus fluoroquinolones | -0.019 | 0.010 | 0.713 | -1.244 to 1.388 |
| Βeta-lactams plus macrolides | -0.552 | -0.021 | 0.549 | -1.623 to 0.435 |
| Non-invasive mechanical ventilation | 0.795 | -0.088 | 1.851 | -1.128 to 2.512 |
| Invasive mechanical ventilation | 1.324 | 0.143 | 0.652 | 0.231 to 3.281 |

Abbreviations: BCa, adjusted bootstrap; CI, confidence interval; ICU, intensive care unit; SE, standard error.
